# Updates for *EHP* Meeting Reports, Grants

**Published:** 2007-11

Beginning in November 2007, *EHP* will fold its Workgroup Report format in with its Meeting Reports, with both to be known hereafter as Meeting Reports. By consolidating these article types and tightening the requirements for their publication, we aim to better meet the needs of our readers with timelier, more substantive reports of important events in the field of environmental health science. All Meeting Reports will be peer reviewed for scientific content and the significance of their scientific and/or policy-related contribution to the field. These short (maximum 5,000 words) synopses of conferences, symposia, workgroup meetings, or workshops should be submitted to *EHP* no later than 9 months after the events they describe, and each should clearly present the event’s objectives, issues, conclusions, and recommendations, as well as background information as a context for the event. Meeting Reports may review existing information, summarize research findings on specific topics, and recommend methods, courses of action, or further research needs for the scientific community. *De novo* data and participant lists are not allowed in Meeting Reports.

In other news, the Grants, Fellowships, & Awards section is now a web-only feature of *EHP*. This will allow us to update the section more frequently and include more information, heightening the section’s value to *EHP* readers.

